# Data-driven modeling of subharmonic forced response due to nonlinear resonance

**DOI:** 10.1038/s41598-024-77639-5

**Published:** 2024-10-29

**Authors:** Joar Axås, Bastian Bäuerlein, Kerstin Avila, George Haller

**Affiliations:** 1 Institute for Mechanical Systems, ETH Zürich, Leonhardstrasse 21, 8092 Zürich, Switzerland; 2 Institute of Physics, University of Oldenburg, Ammerländer Heerstrasse 114-118, 26129 Oldenburg, Germany; 3 ForWind-Center for Wind Energy Research, Küpkersweg 70, 26129 Oldenburg, Germany

**Keywords:** Mechanical engineering, Applied mathematics

## Abstract

Complex behavior in nonlinear dynamical systems often arises from resonances, which enable intricate energy transfer mechanisms among modes that otherwise would not interact. Theoretical, numerical and experimental methods are available to study such behavior when the resonance arises among modes of the linearized system. Much less understood are, however, resonances arising from nonlinear modal interactions, which cannot be detected from a classical linear analysis. Academic examples of such phenomena have been known, but no systematic method has been developed to detect and model nonlinear resonant interactions purely from numerical or experimental data. Here, we develop such a data-driven methodology that identifies nonlinear resonant response on low-dimensional spectral submanifolds (SSMs) of the dynamical system. Our approach is generally applicable to nonlinear resonances, but we specifically focus here on one particular behavior: subharmonic response in forced nonlinear systems without any resonance among the linearized frequencies of the unforced system. We first illustrate analytically how such a response is born out of a nonlinear resonance hidden in the conservative limit of the system. We then show how this effect can be identified and modeled purely from data. As our main example, we isolate and model previously unexplained response patterns in fluid sloshing experiments.

## Introduction

Resonances are an integral feature of dynamical systems at all scales in nature, from particles to planets. Linear resonances occur at a fixed point of an oscillatory system with two or more rationally commensurate eigenfrequencies. Under periodic forcing, such resonances can result in energy transfer^1^ and periodic and quasiperiodic orbits^2,3^. For higher amplitudes in nonlinear systems, the eigenfrequencies generally deviate from their linear limit. This can introduce nonlinear resonances. In systems as diverse as celestial mechanics^4^, thin beam and shell vibrations^5,6^, nonlinear energy sinks^7^, micro- and nano-resonators^8^, and population dynamics^9^, nonlinear resonances give rise to periodic, quasiperiodic, and chaotic behavior.

When confronted with nontrivial response to periodic forcing of a dissipative system, the experimentalist first seeks to identify linear resonances by comparing eigenfrequencies. Should there be no obvious indication of a linear resonance, the phenomenon may be the result of a nonlinear resonance. As there is no established global analytical method for its detection, however, a nonlinear resonance is considerably harder to identify.

In this paper, we introduce a theoretical scenario that explains the emergence of such nonlinear resonances, develop a general methodology that enables their detection, and present new experimental data to which we apply our method. Our methodology is based on recent advanced Melnikov methods^10^ and data-driven spectral submanifold (SSM) modeling^11^. Specifically, Melnikov methods can track periodic orbits of high-dimensional conservative systems under small damping and forcing, while SSMs offer a mathematically justified model reduction scheme for nonlinear systems.

A (primary) SSM is defined as the smoothest invariant manifold tangent to an eigenspace of the linearized system at a fixed point^12^. Each eigenspace with eigenvalues not in linear resonance with eigenvalues outside that eigenspace has a unique SSM of the same dimension. We call such eigenspaces nonresonant. The SSM is as smooth as the system itself and can be viewed as a nonlinear normal mode^13^. The reduced dynamics on the two-dimensional SSM emanating from an oscillatory eigenspace yields a nonlinear frequency and nonlinear damping for the dominant dynamics. Combining several such nonlinear modes into a higher-dimensional SSM in turn allows us to study nonlinear resonances between those frequencies. Generally, an infinite family of secondary SSMs also exists for each spectral subspace. Members of this family are also tangent to the same eigenspace at the fixed point but have lower differentiability than the primary SSM^12^. Due to its smoothness, each SSM can be computed as a Taylor expansion when the equations of motion are known^15^. For a system known only from experimental data, we can obtain a model of a normally attracting SSM from the part of the data that lies near the SSM^11^. Data-driven techniques developed for this purpose^16,17^ return the best approximation of the geometry and reduced dynamics of the SSM subject to the trajectories used for training. For these methods to apply, we assume that the system dynamics are smooth. Extensions of SSMs to non-smooth systems are discussed in Ref. ^18^.

**Figure 1:**
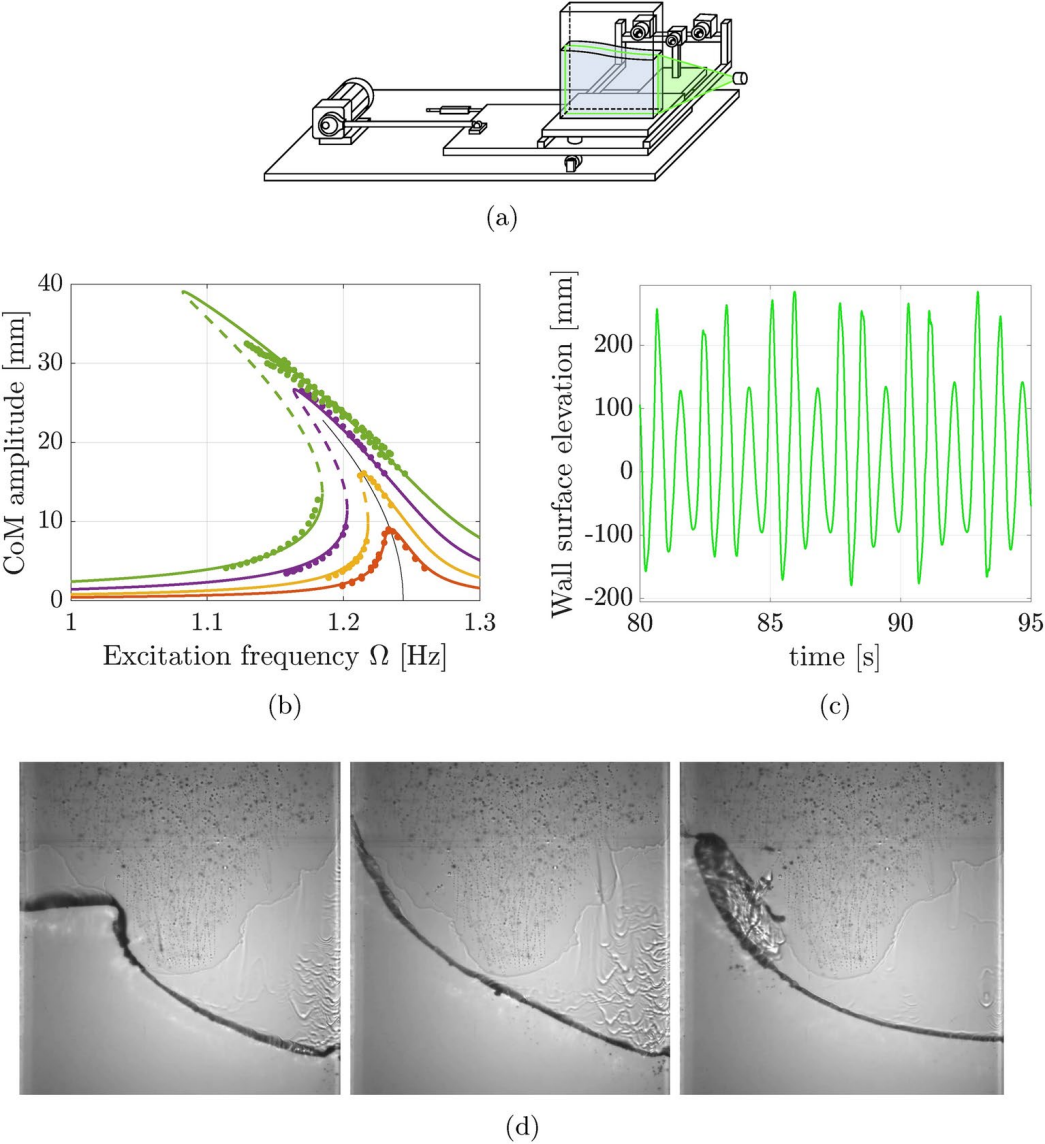
(**a**) Experimental setup of a water tank horizontally excited by a motor^14^. The cameras attached to the rig record the surface profile. (**b**) Previous SSM modeling of sloshing forced response is only accurate up to moderate excitation amplitudes. At the highest amplitude (green top branch), the water response becomes multiharmonic, not captured by a 2D SSM model^11^. (**c**) The surface elevation at the left tank wall at the highest excitation amplitude has a period three times longer than that of the forcing. (**d**) Snapshots of the water tank at three subsequent forcing periods show a recurring pattern of collapse in every third cycle.

An experimental example of nonlinear resonances is liquid sloshing, relevant in road transportation of fluids^19^, spacecraft fuel storage^20^, and maritime cargo handling^21^. For the canonical case of a rectangular tank filled with a deep layer of water subject to harmonic horizontal excitation (Fig. 1a), a softening sloshing response was reported in Ref. ^14^. This response was well captured by nonlinear local models (Fig. 1b), but above a given forcing amplitude, a puzzling period-three orbit appeared (Fig. 1c).

This subharmonic response, where every third forcing cycle produced a different wave shape (Fig. 1d), was not captured by reduced-order models trained on near-equilibrium data^11,22^. Indeed, the system had no relevant linear resonances to explain the response. Here, by applying our data-driven method, we demonstrate that this complicated behavior originates from a nonlinear resonance between the first two nonlinear modal frequencies.

Our contributions are threefold. First, we propose a previously undiscussed mechanism for subharmonic response arising from nonlinear resonances of the conservative limit of a forced-damped system. Second, we illustrate on a simple example, using the mentioned recent advanced Melnikov methods, that this mechanism does in fact exist. Third, we present data from sloshing experiments and use, for the first time, data-driven SSM reduction directly from forced data to uncover a nonlinear resonance. More generally, we obtain a way to approximate the conservative core of a weakly dissipative system that is only known from experimental data.

In summary, we offer a method to identify the source of unexpected, multi-period response as nonlinear resonances. Our approach is generally applicable as long as the system is close to a (not necessarily integrable) conservative limit.

## Results

### Subharmonic response due to nonlinear resonances

For $n\ge 2$, consider an $n$-degree of freedom Hamiltonian system subject to a small, forced-damped perturbation as

1 $$\begin{aligned} \dot{\varvec{x}} = \varvec{J}\varvec{\nabla }H(\varvec{x}) + \varepsilon \varvec{f}(\varvec{x}, \Omega t), \quad \varvec{x} \in \mathbb {R}^{2n}, \end{aligned}$$

with Hamiltonian ${H:\mathbb {R}^{2n}\rightarrow \mathbb {R}}$, a symplectic matrix ${\varvec{J}\in \mathbb {R}^{2n\times 2n}}$, and a time-periodic, nonconservative perturbation ${\varvec{f}:\mathbb {R}^{2n}\times \mathbb {S}^1\rightarrow \mathbb {R}^{2n}}$ such that the right-hand side is $C^{1}$. Physically, the perturbation $\varvec{f}$ corresponds to the effects of dissipation and periodic forcing. Given the forcing frequency $\Omega$, we define the period $T=2\pi /\Omega$ and the Poincaré map $\varvec{P}_{\varepsilon }:\mathbb {R}^{2n}\rightarrow \mathbb {R}^{2n}$ as

2 $$\begin{aligned} \varvec{P}_{\varepsilon }: \varvec{x}_0 \mapsto \varvec{x}(T, \varvec{x}_0, \varepsilon ). \end{aligned}$$

A $k$-periodic orbit of system (1) is a fixed point for the $k$th iterate $\varvec{P}_{\varepsilon }^{k}$ of the Poincaré map solving the equation

3 $$\begin{aligned} \varvec{R}(\varvec{x}, \varepsilon , k) := \varvec{P}_{\varepsilon }^{k}(\varvec{x}) - \varvec{x} = \varvec{0}. \end{aligned}$$

Assume that on a compact domain $\mathcal {C}\in \mathbb {R}^{2n}$ and for a given positive integer $k$, we have $\varvec{R}(\varvec{x}, 0, k) \ne \varvec{0}$. Then, by the continuity of $\varvec{P}_{\varepsilon }$ in $\varepsilon$ and $\varvec{x}$, Eq. (3) has no solution for small enough $\varepsilon >0$ either. Therefore, to locate forced response for arbitrarily small forcing and damping, we must seek a corresponding fixed point $\varvec{x}_{0,k}\in \mathcal {C}$ of $\varvec{P}_{0}^{k}(\varvec{x})$ satisfying

4 $$\begin{aligned} \varvec{R}(\varvec{x}_{0,k}, 0, k) = \varvec{0}. \end{aligned}$$

By definition, such a point $\varvec{x}_{0,k}$ must be a period-$k$ point of the Hamiltonian Poincaré map $\varvec{P}_{0}(\varvec{x})$.

Thus a periodic orbit of a weakly periodically forced and damped system cannot appear out of nothing; the system’s conservative limit must have a nearby closed orbit of similar period. In generic multi-degree of freedom Hamiltonian systems, periodic behavior is rare. We only know of two scenarios in which nontrivial periodic orbits invariably arise: near elliptic fixed points and at nonlinear resonances^23,24^.

#### Survival of periodic orbits near a fixed point

**Figure 2:**
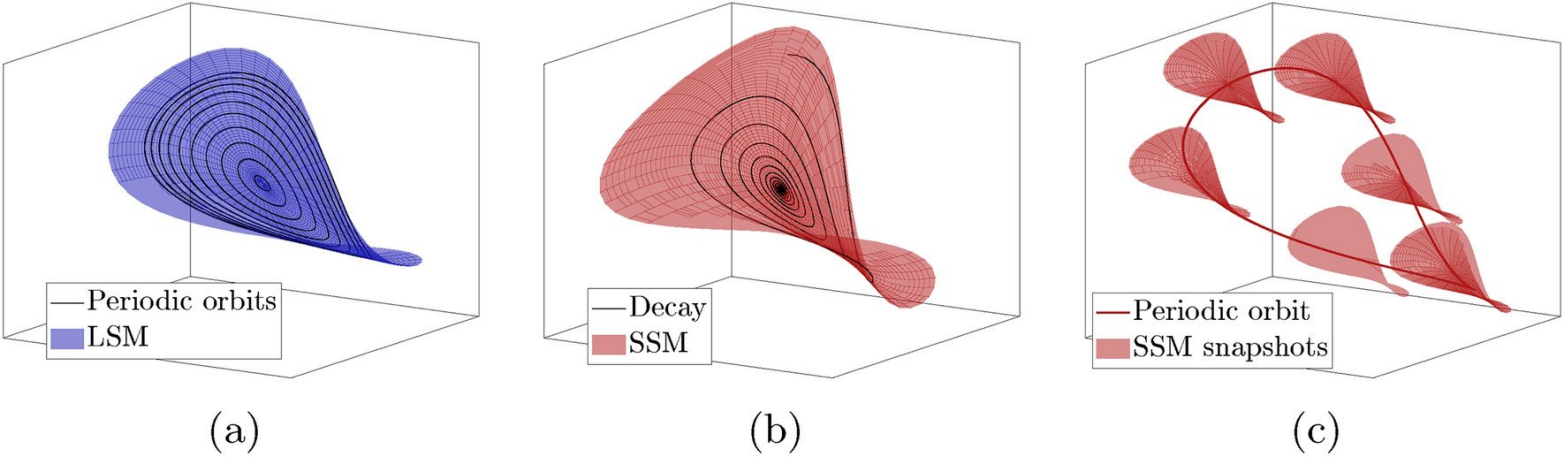
Nonlinear modal dynamics in conservative, damped and forced-damped systems. (**a**) Near a nonresonant elliptic fixed point, a Hamiltonian system admits unique Lyapunov subcenter manifolds (LSMs) foliated by periodic orbits. (**b**) Under a small autonomous nonconservative perturbation, the LSM perturbs into a spectral submanifold (SSM) containing decaying trajectories. (**c**) Additional small periodic forcing creates a nearby periodic orbit, which itself has an attached time-periodic SSM containing the trajectories with the slowest decay to the periodic orbit.

In the first scenario, small-amplitude periodic orbits arise in one-parameter families perturbing from nonresonant eigenmodes of the linearized system at an elliptic fixed point. Represented in Fig. 2a, such families are called Lyapunov subcenter manifolds (LSMs)^25^ or conservative nonlinear normal modes^13,26^.

When such a conservative system is subject to small damping, its LSMs perturb into two-dimensional autonomous spectral submanifolds (SSMs)^27^ (Fig. 2b). Due to their intimate relationship with LSMs and linear modes, SSMs offer a rigorous generalization of nonlinear normal modes to nonconservative systems.

Under further addition of small time-periodic forcing to such a dissipative system, the fixed point perturbs into a small-amplitude periodic orbit^10^. The nearby dynamics are captured by a time-periodic SSM attached to the orbit^28–30^. As illustrated in Fig. 2c, this SSM is anchored to and slides along the periodic orbit. In the limit of zero dissipation and no forcing, the SSM converges to the LSM^10^.

Certain periodic orbits of the LSM limit may also survive within the corresponding SSM under weak forcing and light damping. This can happen if during one period of an LSM periodic orbit, the total energy balance between forcing and damping is zero^10^. However, such periodic, weakly forced-damped response is always close to an LSM of the conservative limit^27,28,31^. Therefore, if an observed limit cycle is not captured by a nearby two-dimensional SSM, it could not have originated from an LSM.

**Figure 3:**
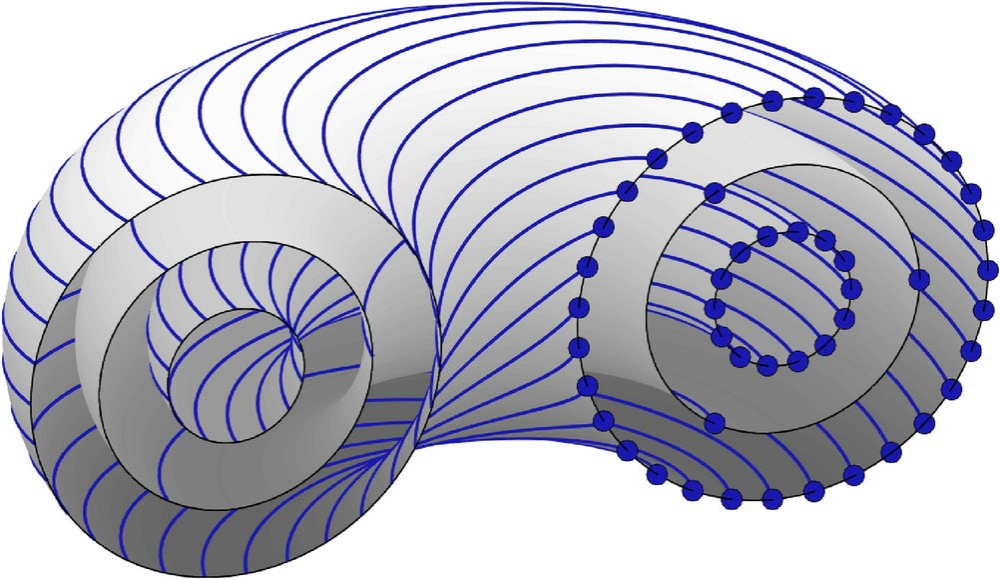
The phase space of a nondegenerate integrable $n$-degree of freedom system is mostly foliated by invariant $n$-tori containing quasiperiodic orbits. Tori with resonant frequencies are instead filled by infinitely many periodic orbits, such as the three-periodic orbit illustrated on the middle torus. Upon every third revolution about the vertical axis, this orbit closes up with itself.

#### Survival of periodic orbits at a nonlinear resonance

In the second scenario, periodic orbits in near-integrable Hamiltonian systems arise due to nonlinear resonances. These periodic orbits appear in resonance gaps created in the destruction of near-resonant quasiperiodic $n$-tori under the addition of non-integrable Hamiltonian perturbations. More specifically, the phase space of a generic integrable system is foliated by invariant tori, most of which are quasiperiodic. If the nonlinear frequencies come in resonance, however, all orbits on the corresponding torus are periodic, as represented by Fig. 3. If the period of one oscillation is $T$ and the second oscillation has period $\frac{k}{m}T$ with minimal integers $m,k\in \mathbb {N}^+$, the periodic orbit has period $kT$. We give an example of such a Hamiltonian system in the upcoming section.

What happens to these periodic orbits under a small perturbation to the integrable system? A classic result on near-integrable systems is the Kolmogorov-Arnold-Moser (KAM) theory^32–34^. KAM theory relates the phase space structure around an elliptic nonresonant fixed point of a generic Hamiltonian or reversible system to its integrable limit. Under appropriate nondegeneracy conditions on the frequency distribution, KAM theory guarantees the survival of most quasiperiodic tori^35^. While resonant and nearly resonant tori are wiped out, lower-dimensional tori may survive in the resonance gaps that open up between the persisting quasiperiodic tori^36,37^.

For our purposes, the relevant question is the survival of periodic orbits, i.e., the special case of 1D tori. The case of a periodic orbit surviving the nonintegrable perturbation was proven by Poincaré under nondegeneracy conditions on the Hessians of the integrable Hamiltonian and the integral of the perturbation over one period of the orbit^38^. A later theorem by Birkhoff implies that at the breakdown of a generic resonant *n*-torus, at least $2^{n-1}$ periodic orbits survive^23^. Continuation in the energy yields a family of periodic orbits for small perturbations^38^.

The addition of small damping and $T$-periodic forcing to such a surviving resonant periodic orbit could, in principle, result in $kT$-periodic subharmonic forced response. We expect this to happen if the energy input from the forcing is equal to the energy loss from the damping over one period of a $kT$-periodic orbit of the conservative limit. In what follows, we detail under what conditions such a phenomenon can occur.

### Survival of a nonlinear resonant periodic orbit under damping and forcing in an analytical example

We begin with a 2-degree of freedom integrable system and add perturbations in two steps to produce a nonconservative system with a 2-periodic orbit, i.e., a response that has twice the period of the applied forcing. To simplify the algebra, we use the complex-valued near-integrable Hamiltonian

5 $$\begin{aligned} \begin{aligned} H(\varvec{z})&= i\left( \omega _1 z_1\bar{z}_1 + \omega _2z_2\bar{z}_2 + \frac{\beta _1z_1^2\bar{z}_1^2}{2} + \frac{\beta _2z_2^2\bar{z}_2^2}{2}\right) \\&\quad + i\epsilon (\bar{z}_1^3z_2^2 + z_1^3\bar{z}_2^2), \end{aligned} \end{aligned}$$

for $\omega _{j}>0$, $\beta _{j}\ne 0$, generating the dynamical system

6 $$\begin{aligned} \begin{aligned} \dot{z}_1&= \frac{\partial H}{\partial \bar{z}_1} = i\omega _1z_1 + i\beta _1z_1^2\bar{z}_1 + 3i \epsilon \bar{z}_1^2z_2^2,\\ \dot{z}_2&= \frac{\partial H}{\partial \bar{z}_2} = i\omega _2z_2 + i\beta _2z_2^2\bar{z}_2 + 2i \epsilon z_1^3\bar{z}_2, \end{aligned} \end{aligned}$$

along with the complex conjugates of these equations. We choose $\omega _1 = 1$, $\omega _2 = 1.4$, and $\beta _1 = \beta _2 = -0.1$. System (6) is a special case of the general 2:3 resonant Hamiltonian normal form up to 4th order^39^.

It is convenient to pass to polar coordinates for analysis. With $z_1 = \rho _1{\rm e}^{i\theta _1}$, $\bar{z}_1 = \rho _1{\rm e}^{-i\theta _1}$, $z_2 = \rho _2{\rm e}^{i\theta _2}$, and $\bar{z}_2 = \rho _2{\rm e}^{-i\theta _2}$, Eq. (6) becomes

7 $$\begin{aligned} \begin{aligned} \dot{\rho }_1&= 3\epsilon \rho _1^2\rho _2^2\sin (3\theta _1-2\theta _2),\\ \dot{\rho }_2&= -2\epsilon \rho _1^3\rho _2\sin (3\theta _1-2\theta _2),\\ \dot{\theta }_1&= \omega _1 + \beta _1\rho _1^2 + 3\epsilon \rho _1\rho _2^2\cos (3\theta _1-2\theta _2),\\ \dot{\theta }_2&= \omega _2 + \beta _2\rho _2^2 + 2\epsilon \rho _1^3\cos (3\theta _1-2\theta _2). \end{aligned} \end{aligned}$$

Studying first the integrable ($\epsilon =0$) limit, we conclude that all trajectories live on invariant, two-dimensional tori characterized by constant values of $\rho _1$ and $\rho _2$. At amplitudes for which $\dot{\theta }_2/\dot{\theta }_1$ is a rational number, the torus is resonant and its orbits close up. For all other amplitudes, the torus is densely filled with quasiperiodic orbits.

**Figure 4:**
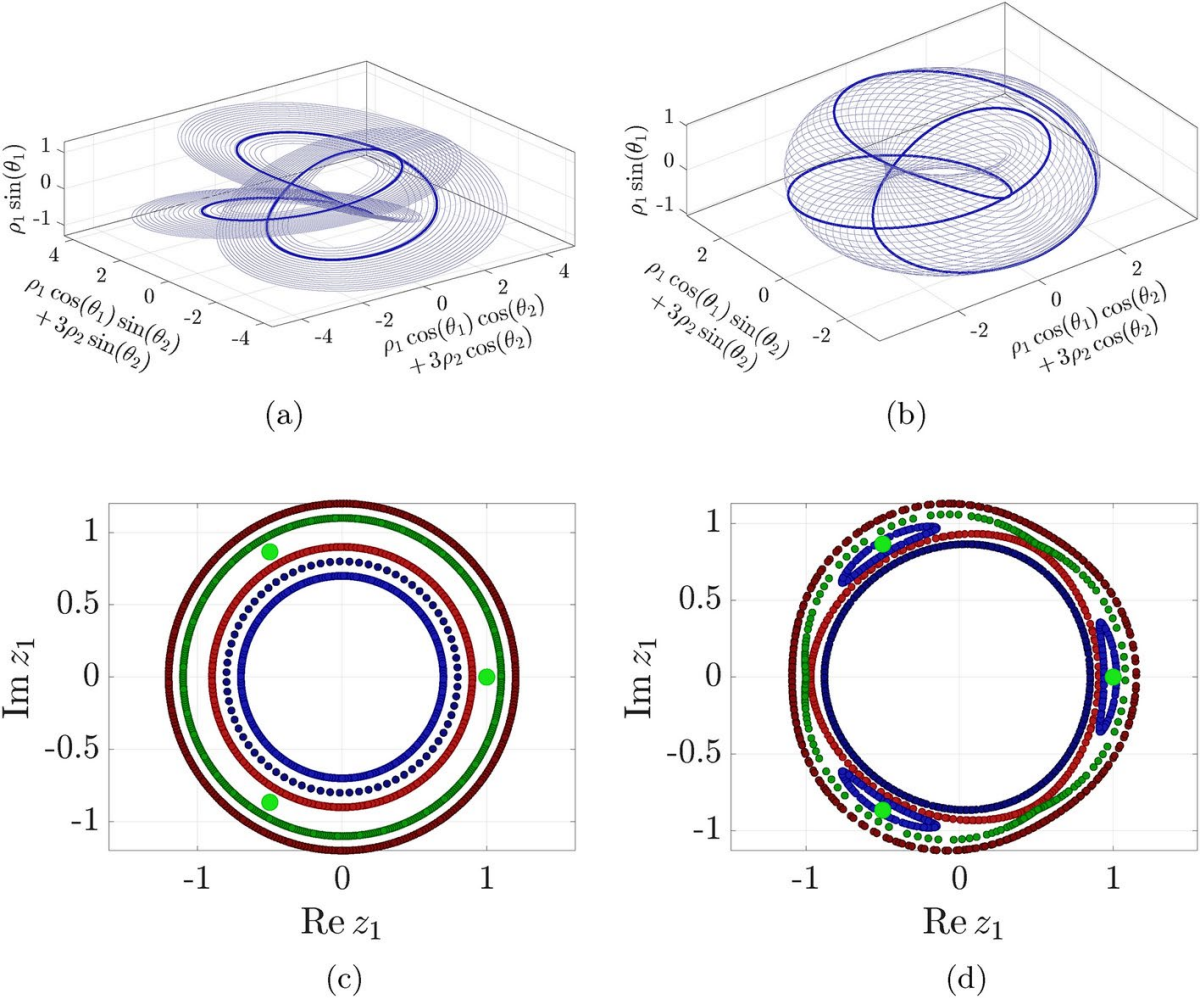
(**a**) The radial family of periodic orbits of the integrable limit ($\epsilon =0$) of system (7) in toroidal coordinates. (**b**) The tangential family of periodic orbits of the same system. This family is destroyed for $\epsilon >0$. (**c**) Taking the intersection at $\theta _2=0$ for the integrable system ($\epsilon =0$) shows the quasiperiodic tori as concentric circles. The three enlarged light green points correspond to one of the periodic orbits on a resonant torus. (**d**) Under the non-integrable perturbation ($\epsilon =10^{-3}$), most quasiperiodic tori persist (dark blue, dark red), but the resonant torus is destroyed and replaced by a spectral gap with pendulum-type dynamics (green, red, blue). The light green points survive as an elliptic periodic orbit.

For example, whenever

8 $$\begin{aligned} \rho _2^2 = (3\omega _1-2\omega _2+3\beta _1\rho _1^2)/2\beta _2, \end{aligned}$$

we obtain $\dot{\theta }_2/\dot{\theta }_1 = 3/2$. Due to this resonance, initial amplitudes satisfying Eq. (8) produce period-two orbits that close up after two revolutions in $\theta _1$. Figure 4a highlights such an integrable periodic orbit starting at $\rho _1=1$, $\rho _2=\sqrt{2}/2$, $\theta _1(0)=\theta _2(0)=0$, also showing other periodic trajectories with initial conditions (8).

In total, Eq. (8) defines a two-parameter family of resonant periodic orbits. Indeed, in geometric terms, starting from one periodic orbit, we can either change the initial condition radially under the constraint (8) or tangentially along the torus to obtain another nearby 2-periodic orbit. Figure 4b shows the tangential component of the family.

To illustrate the dynamics in a neighborhood of this torus, we fix $\rho _2=\sqrt{2}/2$, pick initial conditions $\rho _1\in \{0.7,0.8,\dots ,1.2\}$, and take the $\theta _2$-based first-return map of the resulting trajectories in Fig. 4c. Specifically, each time a trajectory crosses the $\theta _2=0$ section, we register its position and plot it as a dot in the $z_1$-plane. An initial condition on the resonant torus $\rho _1=1$ results in only three points (shown in light green), as the trajectory closes up every third revolution.

Having established the existence of a two-parameter family of 2-periodic orbits in the integrable limit, we turn to the near-integrable system (7) for $\epsilon =10^{-3}$. In this case, 2-periodic orbits appear at initial conditions satisfying

9 $$\begin{aligned} \rho _2^2 = \frac{3\omega _1-2\omega _2+3\beta _1\rho _1^2-4\epsilon \rho _1^3}{2\beta _2-9\epsilon \rho _1}, \quad \theta _2 = 3\theta _1/2. \end{aligned}$$

Due to the additional angle condition arising from the $\mathcal {O}(\epsilon )$ terms in Eq. (7), the tangential family of orbits (Fig. 4b) is destroyed by the perturbation, but the radial family (Fig. 4a) persists and only moves slightly for small $\epsilon$. Therefore, the resulting non-integrable system now only has a one-parameter family of 2-periodic orbits in the gap arising near the destroyed resonant torus.

An intricate local structure with pendulum-type dynamics is formed in the resonance gap around the destroyed torus^37^. This can be seen in Fig. 4d, which shows again the first-return map for a set of initial conditions but for non-zero $\epsilon =10^{-3}$.

We now take the second step in our analysis and add a small nonconservative periodic perturbation to system (6) in the form

10 $$\begin{aligned} \begin{aligned} \dot{z}_1&= \frac{\partial H}{\partial \bar{z}_1} + \varepsilon (f\cos {\Omega t} - cz_1), \\ \dot{z}_2&= \frac{\partial H}{\partial \bar{z}_2} - \varepsilon cz_2, \end{aligned} \end{aligned}$$

with $\varepsilon =10^{-3}$, $c=0.2$, $f=1$, and $\Omega = 2\pi /T$, where $2T$ is the period of the near-integrable periodic orbit starting at $\rho _1=1$. Our objective is to predict whether any of the conservative 2-periodic orbits survive under this additional nonconservative perturbation. Of practical interest is the case in which such a surviving orbit is stable and hence directly observable. Following Refs. ^10,40^, we answer these questions by a recently developed Melnikov-type analysis of the perturbation. Unlike classic Melnikov-type methods (see, e.g., Ref. ^41^), the version applied here does not require the unperturbed system to be integrable.

Applied to this example, the generalized Melnikov function for a periodic orbit $\varvec{z}(t)$ subject to a nonconservative $T$-periodic perturbation $\varepsilon \varvec{g}(\varvec{z},t)$, such as the one applied in Eq. (10), reads

11 $$\begin{aligned} M(s) = \int _0^{2T} \varvec{\nabla }H(\varvec{z}(t+s))^\top \varvec{g}(\varvec{z}(t+s), t)\,dt. \end{aligned}$$

This function describes the work performed by the force minus the energy dissipated by the damping over a periodic orbit of the limiting conservative system. A conservative periodic orbit corresponding to a transverse zero $s_0$ of *M*(*s*), (${M(s_0)=0}$, ${M'(s_0)\ne 0}$), is proven to survive if it is part of a one-parameter family of periodic orbits^10^.

**Figure 5:**
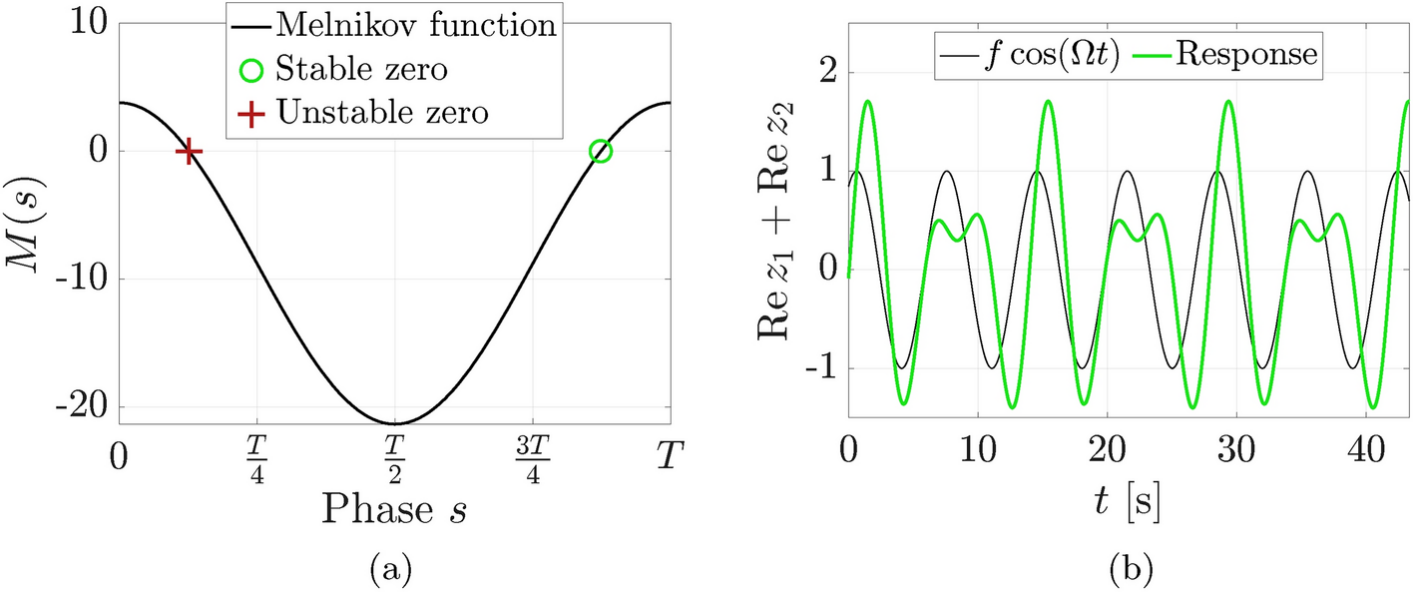
(**a**) The Melnikov function for the forced-damped perturbation has two zeros, predicting two periodic orbits in the nonconservative system (10). (**b**) The resulting forced-damped stable 2-periodic orbit has twice the period of the applied forcing as predicted and originates from the integrable system.

This is formulated in terms of *m*-normality, a condition on the monodromy matrix, i.e., the linearized first-return map around the orbit^10^. In particular, for the present example, the double $+1$ eigenvalue of this map must have geometric multiplicity one. We verify this condition numerically for the $\rho _1=1$ member of the family (9). To this end, we choose initial conditions in a small neighborhood of the periodic orbit, integrate them over one period, and fit a linear map to estimate the eigenspaces of the monodromy matrix.

Further, following Ref. ^10^, we compute and plot the Melnikov function in Fig. 5a and find that it has two transverse zeros. We conclude that at least two nearby periodic orbits must exist in the perturbed system for small enough forcing and damping.

Following the theorems proven in Ref. ^40^ for typical dissipation, if the conservative periodic orbit is elliptic, and the period of the orbit changes locally within the family, one of the Melnikov zeros predicts an asymptotically stable orbit and the other zero an unstable orbit. We verify these conditions numerically, again employing our estimate of the monodromy matrix.

Indeed, when we integrate an initial condition on the conservative periodic orbit for the perturbed system with the forcing phase coinciding with a zero of the Melnikov function, the trajectory converges to a nearby 2-periodic orbit under forcing and damping. Figure 5b shows that, as expected, this response has twice the period of the applied forcing.

In summary, a nonlinear resonance in the conservative limit of a weakly forced-damped system gives rise to a stable subharmonic forced response if the underlying resonant periodic orbits form an elliptic one-parameter family, the period varies within the family near the orbit, and the Melnikov function has transverse zeros. The conservative core must therefore be non-integrable, because periodic orbits of integrable systems appear in multi-parameter families.

### Data-driven confirmation in the sloshing example

The oscillatory motion of liquid in containers can exhibit a wide range of nonlinear behavior. In the linear limit, the free liquid motion can be decoupled into sloshing modes, corresponding to different wave shapes and eigenfrequencies^42^. Taking nonlinearities and dissipation into account, these sloshing modes perturb into SSMs. While nonlinear effects are evident already under small harmonic forcing, larger excitation activates several coupled modes giving rise to a range of complex wave motions^42–44^.

Data-driven SSMs have repeatedly shown promise as sloshing models. These methods are applied to identify the SSM tangent to the main sloshing modes from decaying data and subsequently predict the system’s response to periodic forcing by adding a time-dependent term to the reduced dynamics. First, Ref. ^11^ captured the forced response of the slowest sloshing mode in a water tank using a 2D SSM trained only on the liquid’s extracted center of mass signal. This analysis was subsequently extended to the full surface profile for training a refined 2D model^22^. Recently, Ref. ^45^ discovered and exploited a mathematical structure in delay-embedded spaces to identify a 6D SSM, corresponding to the nonlinear extension of the three major modes in more complex wave shapes. Here, we examine subharmonic forced response occurring at even higher excitation amplitudes.

We study new data from experiments detailed in Ref. ^14^ and illustrated in Fig. 1a. A rectangular tank is mounted on a horizontally moving platform and partially filled with water. The container is narrow, allowing us to treat the wave motion as two-dimensional. We excite the platform harmonically and film and extract the resulting water surface profile along the tank width. For small excitations, the sloshing wave shape changes depending on forcing level and frequency, but the response is always monoharmonic.

At a slightly higher forcing amplitude near resonance, however, the sloshing motion becomes subharmonic, as every third wave peak collapses at the tank wall (Fig. 1d). Consequently, unlike the low-amplitude wave shapes, this response period is three times longer than the forcing period.

To examine the unforced dynamics, we excite the system to a 3-periodic state and then turn off the motor. The resulting decaying motion is multimodal, suggesting that the forced phenomenon is the result of an interplay of several sloshing modes^45^. An explanation of this sustained motion requires a mechanism by which energy is transferred from the first to the second mode. Typically, modal energy transfer occurs at resonances^1^, but the ratio of the two first linear eigenfrequencies is $\sqrt{2}$, an irrational number. As shown in our analytical example, however, another source of sustained multimodal forced response is a nonlinear resonance.

The first step in our analysis is based on Ref. ^45^. We take three trajectories of decaying surface profile data, two starting at the period-three state and one at a wave-breaking state, and identify an SSM corresponding to the two slowest modes using the open source package fastSSM^17^. In this analysis, we leverage analytical knowledge of the eigenfrequencies and sloshing mode shapes to find the SSM tangent space, as explained in Ref. ^45^. We then compute the reduced dynamics on the SSM in the polar normal form, which yields instantaneous modal frequencies $\dot{\theta }_1$ and $\dot{\theta }_2$ as functions of the amplitudes $\rho _1$ and $\rho _2$. While we cannot measure the Hamiltonian limit of the system, we infer the conservative limit of the SSM-reduced dynamics by letting $\dot{\rho }_{j}$ approach zero in our damped model. As our model predicts small dissipation in the range of the data, it follows that the dissipative frequencies lie close to their conservative counterparts.

**Figure 6:**
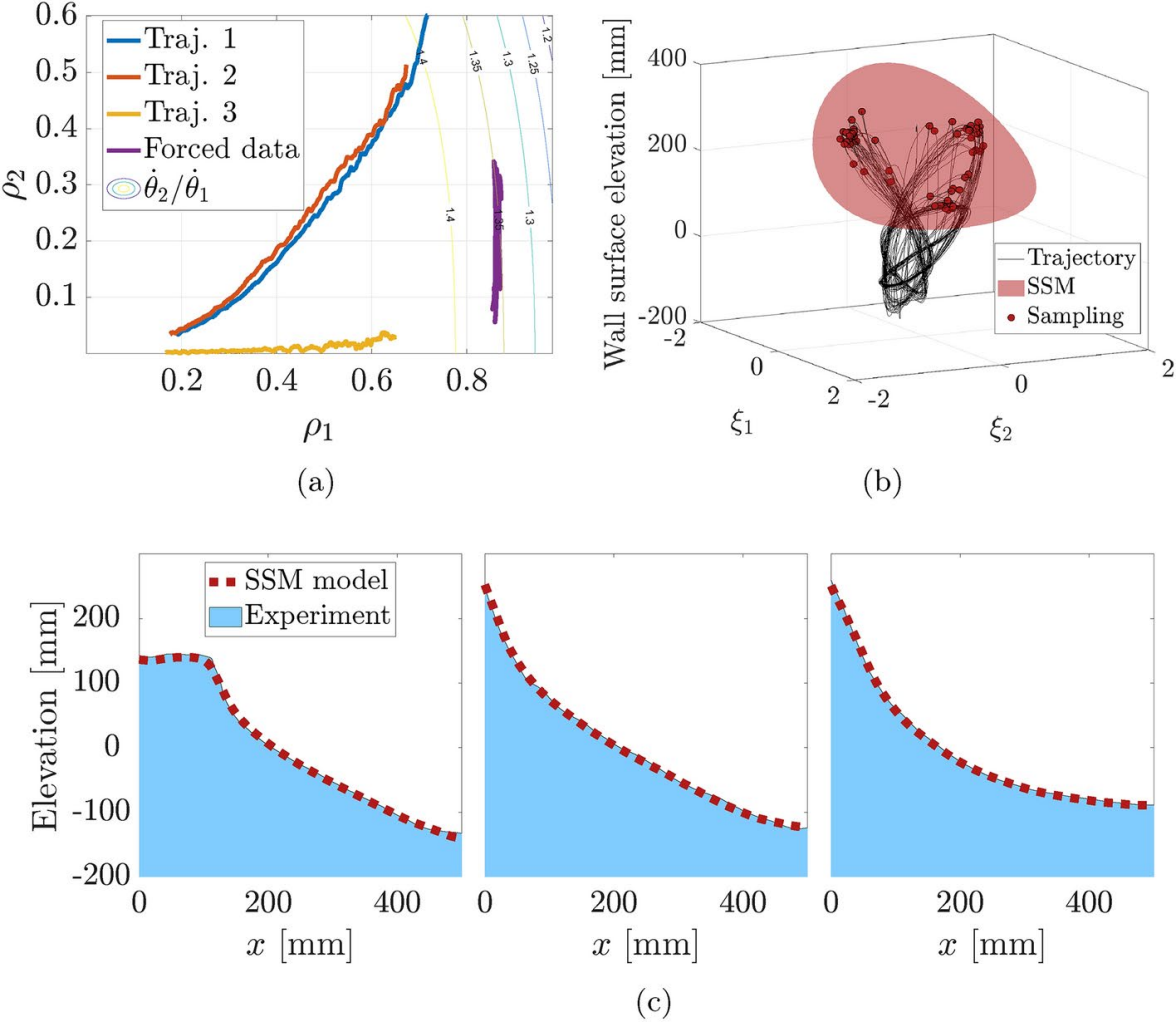
(**a**) Autonomous 4D SSM model of the nonlinear modal frequency ratio $\dot{\theta }_2/\dot{\theta }_1$ as a function of amplitudes, along with decaying trajectories (blue, red, yellow). This ratio reaches 4/3 in the regime of the 3-periodic response (purple), signaling that the response originates from this nonlinear 4:3 resonance, as in our analytical example. (**b**) The periodical samples (red dots) of a forced continuous trajectory (black) lie on a 2D SSM (red). In the reduced coordinates $\xi _1, \xi _2$ parametrizing the SSM, trajectories converge onto a three-periodic point. (**c**) SSM prediction of test data for three subsequent periods shows accurate prediction of the sloshing 3-periodic orbit.

We plot the ratio of the instantaneous frequencies $\dot{\theta }_2/\dot{\theta }_1$ in Fig. 6a along with the three decaying trajectories and one forced 3-periodic trajectory. In the range of the 3-periodic data, the predicted frequency ratio is near $\frac{4}{3}$. In conclusion, there is a strong indication that the three-periodic response has arisen as a perturbation from a 4:3 resonant torus of the conservative system.

We now train an SSM-based polynomial model to predict this sustained subharmonic motion. To this end, we focus on SSMs in the Poincaré map, taking a snapshot of the system once every forcing period.

Out of four measurements sampled with a timestep $\Delta t=0.033$ s, we use one trajectory for training and three for testing. We initialize each measurement at a different forcing frequency, $[0,\,1.05,\,1.2,\,1.23]$ Hz. At the start of the experiment, we then change the frequency to 1.145 Hz, which causes the flow to eventually converge to a 3-periodic orbit. As we wish to model not only the 3-periodic response, but also its stability type and nearby dynamics, we include transient data not yet converged to the attracting periodic orbit.

Based on the recommendations in Ref. ^45^, we delay-embed the data with embedding dimension 22 and timelag $7\Delta t$. This reproduces a periodic orbit in a $30\,932$-dimensional observable space consisting of 22 delays of surface profile measurements at $1\,406$ points along the tank width.

Taking the Poincaré map would normally imply discarding the data between the samples, but due to the delay embedding, each section of the map contains a short time series of the signal. In effect, we are taking the Poincaré map at several phases simultaneously, and consequently reduce the data loss. Therefore, we also find that the choice of phase for the sampling has little effect on the results.

A 2nd order, 2D SSM in the Poincaré map, visualized in Fig. 6b, with 3rd order reduced dynamics reliably captures the period-3 response and close transients approaching it. This figure includes the continuous trajectory in black and the sampled red points, which are confined to the SSM. In the local coordinates on this SSM, we see a clear triangular structure for the asymptotic reduced dynamics.

**Table 1:** Normalized errors^11^ for the Poincaré map SSM model predictions on each transient period-3 trajectory.

Trajectory	Type	Error
1	Test	5.8 %
2	Test	5.9 %
3	Test	4.4 %
4	Training	5.4 %

We find that the dynamics model is accurate and predictive; we only need to train on one of the transient trajectories to predict the time series of all four trajectories. After we lift the predicted trajectories to the full observable space via the SSM parametrization, the normalized mean trajectory error^11^ in the prediction of the full surface profile is 5.4 %. The error measures for each trajectory is shown in Table 1. Figure 6c shows the prediction from this 2D model for the three subsequent snapshots of the surface profile for trajectory 1 in the test data.

The SSM-reduced model also predicts an unstable fixed point at the center of the triangle, from which this 2D unstable SSM emanates. This unstable 1-periodic orbit is likely a remnant of the LSM emanating from the first mode.

## Discussion

We have shown how subharmonic response in oscillatory nonlinear dynamical systems subject to damping and periodic forcing can arise as a consequence of nonlinear resonances in their conservative limits. In particular, we illustrated on an example how nonlinearly resonant periodic orbits persist from the integrable limit of a forced-damped system. We then used the same mechanism to explain a high-amplitude three-periodic response observed in sloshing experiments.

Our analytical example highlights under what conditions subharmonic response due to a nonlinear resonance can appear under small damping and periodic forcing. In particular, the conservative limit must contain a generic one-parameter family of resonant periodic orbits.

Furthermore, these periodic orbits must be elliptic to remain stable after the nonconservative perturbation. Under these conditions, a nonconservative sufficently small periodic perturbation produces a stable subharmonic orbit if a recently developed Melnikov function has an appropriate transverse zero for some forcing phase. This survival is akin to how LSMs, which are one-parameter families of periodic orbits arising from 2D modal subspaces of a conservative linearized system, perturb into the frequency response curve of harmonically excited dissipative mechanical systems.

In the liquid sloshing experiments analyzed here, we explained the observed three-periodic motion as one originating from a nonlinear resonance of the same kind as in our analytical example. We viewed the response as a perturbation of a conservative limit, in which we were seeking a nonlinear resonance. Upon closer inspection of the dynamics on a 4D SSM constructed from the experimental data, we indeed found a nearby 4:3 resonance between nonlinear frequencies. This perturbative argument provides an explanation for the subharmonic response that remained uninterpretable in previous studies only concerned with linearized frequencies.

Our methodology is general enough to investigate nonlinear resonances in any weakly periodically forced and damped system exhibiting subharmonic response. Therefore, we expect it to apply to, for example, weakly damped structures, nonlinear energy sinks, and other surface wave settings.

## Methods

Here, we present a new method to identify SSMs from data in the Poincaré map.

### Spectral submanifolds in maps

Consider a nonlinear dynamical system of class $\mathcal {C}^{l}$, where $l\in \{\mathbb {N}^+,\infty ,a\}$, *a* denoting analyticity, with periodic forcing in the form

12 $$\begin{aligned} \dot{\varvec{x}} = \varvec{A}\varvec{x} + \varvec{g}(\varvec{x}) + \varvec{f}_{\rm{ext}}(\Omega t, \varvec{x}), \end{aligned}$$

where $\varvec{x} \in \mathbb {R}^n$ are coordinates in the full phase space, $\Omega > 0$ denotes the forcing frequency, $\varvec{g}: \mathbb {R}^n\rightarrow \mathbb {R}^n$ represents the autonomous nonlinear terms and $\varvec{f}_{\rm{ext}}$ expresses the periodic forcing, mapping from $\mathbb {T}^1 \times \mathbb {R}^{n} \rightarrow \mathbb {R}^{n}$.

Let us denote the flow map of the system by $\varvec{F}^t_{t_0}(\varvec{x}_0) := \varvec{x}(t, t_0, \varvec{x}_0)$, with $\varvec{x}(t, t_0, \varvec{x}_0)$ denoting the trajectory of (12) starting from $\varvec{x}_0$ at time $t_0$. We further denote one forcing period as $T= 2\pi /\Omega$. Now let us fix $t_0$ and sample the system with period $T$ as

$$\begin{aligned} \varvec{x}_{j+1} = \varvec{x}(t_0+(j+1)T) = \varvec{F}^{t_0+(j+1)T}_{t_0}(\varvec{x}_0) = \varvec{F}^{t_0+T}_{t_0}(\varvec{x}_{j}). \end{aligned}$$

We assume a periodic orbit in the continuous system, which in the Poincaré map corresponds to a fixed point $\varvec{x}^{0}$. Then, we can rewrite (12) as an autonomous map in the form

13 $$\begin{aligned} \varvec{x}_{j+1} = \varvec{x}^{0}+ \varvec{B}(\varvec{x}_{j}-\varvec{x}^{0}) + \varvec{G}(\varvec{x}_{j}-\varvec{x}^{0}), \end{aligned}$$

with $\varvec{B}\in \mathbb {R}^{n\times n}$ a matrix describing the linear part of the system around the fixed point and $\varvec{G}: \mathbb {R}^n\rightarrow \mathbb {R}^n$ denoting the higher-order terms.

We assume that $\varvec{x}^{0}$ is a non-degenerate fixed point, i.e., that the absolute value of each eigenvalue of $\varvec{B}\in \mathbb {R}^{n\times n}$ is not equal to one. We take ${d}$ eigenvectors of $\varvec{B}$ and denote their span by $\mathcal {E}$, i.e., a ${d}$-dimensional spectral subspace of $\mathbb {R}^{n}$. In this step, we often choose the ${d}$ slowest eigendirections.

Provided that the eigenvalues in $\mathcal {E}$ are non-resonant with the eigenvalues of $\varvec{B}$ outside of $\mathcal {E}$, the nonlinear system (13) has a unique smoothest, invariant manifold $\mathcal {M}$ tangent to $\mathcal {E}$ at the fixed point, i.e., $T_{\varvec{x}^{0}}\mathcal {M}= \mathcal {E}$^12,28,31^. Following Ref. ^29^, we call $\mathcal {M}$ a spectral submanifold (SSM). In case of a resonance between $\mathcal {E}$ and the rest of the spectrum of $\varvec{B}$, the ${d}$-dimensional SSM does not exist in general, and we must then include the resonant modal subspace into $\mathcal {E}$ to obtain a higher-dimensional SSM. When all eigenvalues of $\varvec{B}$ are stable, the slowest SSM attracts nearby trajectories, which makes it suitable for model order reduction.

The open-source numerical package SSMTool computes SSMs from arbitrary finite-dimensional nonlinear systems^15,46^. More recently, the SSMLearn package was developed to find SSMs in data from nonlinear dynamical systems^11,47^. Here, we apply a new, discrete version of the simplified data-driven SSM algorithm fastSSM introduced by Ref. ^22^.

### Identification of spectral submanifolds from data

Dynamics-based machine learning involves reconstructing an SSM from data and then using a reduced order model on the identfied SSM to predict the full system response^11^. The procedure consists of two steps: manifold geometry detection and reduced dynamics modeling. Here, we present an altered version of fastSSM^17,22^, suitable for discrete rather than continuous systems, to identify the SSM from snaphots in an observable space.

The SSM is parametrized in the graph style, that is, we construct $\mathcal {M}$ as a graph over the spectral subspace $\mathcal {E}$. The data consists of snapshots $\varvec{y}_{k} \in \mathbb {R}^p$ in a $p$-dimensional observable space, sampled with the forcing period $T$. For each trajectory we construct the snapshot matrix $\varvec{Y}\in \mathbb {R}^{p\times N}$ from $N$ snapshots as

14 $$\begin{aligned} \varvec{Y}= \left[ \begin{array}{cccc} \vert & \vert & & \vert \\ \varvec{y}_1 & \varvec{y}_2 & \ldots & \varvec{y}_{N} \\ \vert & \vert & & \vert \\ \end{array}\right] . \end{aligned}$$

Following fastSSM, through singular value decomposition (SVD) on the snapshot matrix $\varvec{Y}-\varvec{y}^{0}$ we obtain a matrix $\varvec{T}\in \mathbb {R}^{p\times {d}}$ whose columns approximately span the SSM tangent space. Note that since the fixed point to which the SSM is attached does not generally lie at the origin, we must first locate its position $\varvec{y}^{0}$ and subtract it from the data before performing SVD. If the fixed point is unknown or unobservable, we approximate it by taking the average of an observed steady state around it. We project each snapshot $\varvec{y}_k$ onto the approximate tangent space and obtain reduced coordinates $\varvec{\xi }\in \mathbb {R}^{{d}}$ as

15 $$\begin{aligned} \varvec{\xi }= \varvec{T}^\dagger (\varvec{y}-\varvec{y}^{0}), \end{aligned}$$

where $(\cdot )^\dagger$ denotes the Moore-Penrose pseudoinverse. We denote by $\varvec{\Xi }\in \mathbb {R}^{{d}\times N}$ the projection of the snapshot matrix onto $\varvec{T}$.

Next, we model the geometry of $\mathcal {M}$ as the graph of a multivariate polynomial of order $m$:

16 $$\begin{aligned} \begin{aligned}&\varvec{y}(\varvec{\xi }) = \varvec{M}\varvec{\xi }^{1:m} + \varvec{y}^{0}, \\ &\varvec{M}= [\varvec{M}_1, \varvec{M}_2, \dots , \varvec{M}_{m}], \quad \varvec{M}_{k} \in \mathbb {R}^{p\times {d}_{k}}, \end{aligned} \end{aligned}$$

where throughout this section ${d}_{1:k}$ denotes the number of ${d}$-variate monomials at orders 1 through $k$ and the superscript in $(\cdot )^{1:k}$ denotes a vector of all monomials from order 1 up to $k$. For instance, if $\varvec{x} = [x_1, x_2]^\top$, then

$$\varvec{x}^{1:3} = [x_1,x_2,x_1^2,x_1x_2,x_2^2,x_1^3,x_1^2x_2,x_1x_2^2,x_2^3]^\top .$$

We compute the manifold parametrization coefficients $\varvec{M}\in \mathbb {R}^{{p}\times {d}_{1:m}}$ with a polynomial regression, i.e.,

17 $$\begin{aligned} \varvec{M}= (\varvec{Y}-\varvec{y}^{0})(\varvec{\Xi }^{1:m})^\dagger . \end{aligned}$$

Similarly, we approximate the reduced dynamics map as an $\mathcal {O}(r)$ polynomial with coefficients $\varvec{R}\in \mathbb {R}^{{d}\times {d}_{1:r}}$, in the form

18 $$\begin{aligned} \varvec{\xi }_{j+1} \approx \varvec{R}\varvec{\xi }^{1:r}_{j}, \quad \varvec{R}= \varvec{\Xi }_{j+1}(\varvec{\Xi }_{j}^{1:r})^\dagger . \end{aligned}$$

In order to apply this method, we must assume that the training data lies sufficiently close to the SSM. We can achieve this by removing initial transients from the input signal^47^. As the SSM tangent to the slowest ${d}$ modes is unique and attracting, generic nearby initial conditions approach it exponentially in forward time, and removal of initial transients therefore ensures relevant training data.

### Delay-embedding invariant manifolds

We delay-embed the continuous dynamical system (12) as opposed to the periodically sampled map (13), and apply the periodical sampling in the observable space. This way, less information is lost in the sampling, which now contains snapshots separated by a phase lag different from the forcing period.

Following the notation in^45^, we define a vector-valued observable $\varvec{\mu }(\varvec{x}(t))$ for the continuous system (12), where $\varvec{\mu }: \mathbb {R}^n\rightarrow \mathbb {R}^q$ is a differentiable function that returns $q$ measured features of the system, such as a set of displacement coordinates. Next, we stack $p$ consecutive measurements separated by a timelag $\tau > 0$ to create an observable space of dimension $pq$. This yields a trajectory in the form $\varvec{y}(t) = \varvec{S}(\varvec{x}(t),t) \in \mathbb {R}^{pq}$, where we define the sampling map

19 $$\begin{aligned} \varvec{S}: \mathbb {R}^n\times \mathbb {R}\rightarrow \mathbb {R}^{pq}, \quad (\varvec{x}, t) \mapsto \left[ \begin{array}{c} \varvec{\mu }(\varvec{x}) \\ \varvec{\mu }(\varvec{F}_t^{t+\tau }(\varvec{x})) \\ \varvec{\mu }(\varvec{F}_t^{t+2\tau }(\varvec{x})) \\ \vdots \\ \varvec{\mu }(\varvec{F}_t^{t+(p-1)\tau }(\varvec{x})) \end{array}\right] . \end{aligned}$$

With delay embedding, invariant sets of (12) in $\mathbb {R}^n\times \mathbb {R}$ are reproduced in the observable space $\mathbb {R}^{pq}$. Assume that $\varvec{x}(t)$ evolves on a ${d}$-dimensional invariant manifold $\mathcal {M}$. Then, Takens’s embedding theorem applied to (12) states that if any component of $\varvec{\mu }$ is generic and no small integer multiple of $\tau$ coincides with the period of any periodic orbit on $\mathcal {M}$, then for

20 $$\begin{aligned} p\ge 2d+1, \end{aligned}$$

the manifold $\mathcal {M}$ has a diffeomorphic copy $\tilde{\mathcal {M}}$ in $\mathbb {R}^{pq}$ via the mapping (19)^48^. For our setting, with Poincaré map period $T$, the periodicity assumption most notably implies that we must have $k\tau \ne T$ for any small $k\in \mathbb {N}$. The manifold $\mathcal {M}$ can be an autonomous SSM, a periodic orbit or invariant torus, or a nonautonomous SSM attached to such a periodic orbit or torus. To apply Takens’s theorem, we must take ${d}$ to mean the dimension of $\mathcal {M}$ in the extended phase space, e.g., for a 2-dimensional SSM attached to a periodic orbit, we must choose at least $p\ge 2(2+1)+1 = 7$, as the periodic orbit has dimension 1.

### Frequency ratio analysis of three-periodic sloshing response

In order to obtain modal frequencies and investigate any nonlinear resonances, we model the continuous dynamics from decaying sloshing measurements on a 4D SSM in a delay-embedded observable space. As we are interested in the continuous dynamics, this method differs from the Poincaré map setting and is instead based fully on the method in Ref. ^45^.

We use three training trajectories to estimate the autonomous damped normal form dynamics. The first two trajectories start at a 3-periodic orbit and the third trajectory starts at a wave-breaking state. We use the surface profile measured at $1\,771$ points sampled with $\Delta t=0.01$ s and delay-embed the data with dimension $p=47$ and timelag $\tau =2\Delta t$. We select these values based on an optimization of the delay-embedded eigenvector separation as detailed in^45^.

The eigenfrequencies and eigenvectors are obtained analytically^14^ and following Ref. ^45^, this determines the eigenvectors at the fixed point in the observable space. We compute these eigenvectors and use the first two pairs for the SSM tangent space.

Then, applying fastSSM, we obtain a 3rd order 4D SSM. Projecting onto this SSM, we identify 5th order reduced dynamics. This dynamics is obtained through regularized polynomial regression on the numerically computed time derivative of the reduced coordinates, with regularization parameter 0.05.

Next, we take this polynomial model of the reduced dynamics and compute its normal form up to 7th order. This model produces a normalized mean trajectory error (NMTE) of 3.85 %^11^ and its normal form dynamics reads

21 $$\begin{aligned} {\begin{array}{ll} \dot{\rho }_{1} =& 0.0030\,{\rho _{1}}^7 -0.0762\,{\rho _{1}}^5\,{\rho _{2}}^2 -0.1017\,{\rho _{1}}^5 +0.0013\,{\rho _{1}}^3\,{\rho _{2}}^4+\\ & +0.0381\,{\rho _{1}}^3\,{\rho _{2}}^2 +0.0550\,{\rho _{1}}^3 -0.0239\,\rho _{1}\,{\rho _{2}}^4 +0.0046\,\rho _{1}\,{\rho _{2}}^2 -0.0598\,\rho _{1}\\ \dot{\theta }_{1} =& -0.0073\,{\rho _{1}}^6 -0.0295\,{\rho _{1}}^4\,{\rho _{2}}^2 -0.3877\,{\rho _{1}}^4 +0.0029\,{\rho _{1}}^2\,{\rho _{2}}^4+\\ & +0.0444\,{\rho _{1}}^2\,{\rho _{2}}^2 -0.6018\,{\rho _{1}}^2 -0.0288\,{\rho _{2}}^4 +0.0181\,{\rho _{2}}^2 +7.7631\\ \dot{\rho }_{2} =& -0.1916\,{\rho _{1}}^6\,\rho _{2} -0.5954\,{\rho _{1}}^4\,{\rho _{2}}^3 +0.2439\,{\rho _{1}}^4\,\rho _{2} -0.0346\,{\rho _{1}}^2\,{\rho _{2}}^5+\\ & -0.0516\,{\rho _{1}}^2\,{\rho _{2}}^3 +0.3386\,{\rho _{1}}^2\,\rho _{2} -0.5179\,{\rho _{2}}^5 -0.5812\,{\rho _{2}}^3 -0.1426\,\rho _{2}\\ \dot{\theta }_{2} =& -0.4568\,{\rho _{1}}^6 -1.3449\,{\rho _{1}}^4\,{\rho _{2}}^2 -2.1013\,{\rho _{1}}^4 -0.0586\,{\rho _{1}}^2\,{\rho _{2}}^4+\\ & -0.3735\,{\rho _{1}}^2\,{\rho _{2}}^2 -0.1641\,{\rho _{1}}^2 -1.2260\,{\rho _{2}}^4 +0.3633\,{\rho _{2}}^2 +11.1226 \end{array} }. \end{aligned}$$

In these equations, $\dot{\rho }_1/{\rho _1}$ and $\dot{\theta }_1$ correspond to the nonlinear damping and frequency of the first mode and $\dot{\rho }_2/{\rho _2}$ and $\dot{\theta }_2$ the damping and frequency of the second mode. We find that the damping remains small and therefore we can approximate the system as a small dissipative perturbation from a conservative limit.

Then, we evaluate the model prediction of the modal frequencies as functions of the amplitudes and compute the ratio $\dot{\theta }_2/\dot{\theta }_1$. We also take forced data on the 3-periodic orbit, project it onto the SSM and pass to normal form coordinates. The resulting phase portrait along with a contour plot of the frequency ratio is shown in Fig. 6a. In this view, we find that the subharmonic response takes place in a $(\rho _1,\rho _2)$ regime where the frequency ratio is approximately $\dot{\theta }_2(\rho _1,\rho _2)/\dot{\theta }_1(\rho _1,\rho _2) \approx 4/3$.

## Data Availability

All data and code discussed in the results presented here is publicly available in the SSMLearn repository at github.com/haller-group/SSMLearn.
